# Supplementary material for: Bayesian refinement of protein structures and ensembles against SAXS data using molecular dynamics
Source: PLoS Comput Biol. 2017 Oct 18;13(10):e1005800. doi: 10.1371/journal.pcbi.1005800 (PMC5662244; doi:10.1371/journal.pcbi.1005800)
Supplement: S1 Archive — (BZ2) [file pcbi.1005800.s011.tar.bz2 › gromacs_4.62_bayesian_saxs_refi/share/top/amber96.ff/forcefield.doc]

AMBER96 protein, nucleic AMBER94 (Kollman et al., Acc. Chem. Res. 29, 461-469, 1996)

********************************************************************

* The original ffamber ports were written by Eric J. Sorin, *

* CSU Long Beach, Dept. of Chem & Biochem, and have now been	*

* integrated with the standard gromacs distribution. *

* (Please don't blame Eric for errors we might have introduced.) *

* For the implementation/validation, please read/cite: *

* Sorin & Pande (2005). Biophys. J. 88(4), 2472-2493. *

* For related material and updates, please consult	*

* http://chemistry.csulb.edu/ffamber/	*

********************************************************************
